# Novel Anti-Fungal d-Laminaripentaose-Releasing Endo-β-1,3-glucanase with a RICIN-like Domain from *Cellulosimicrobium funkei* HY-13

**DOI:** 10.3390/biom11081080

**Published:** 2021-07-22

**Authors:** Lu Bai, Jonghoon Kim, Kwang-Hee Son, Dong-Ha Shin, Bon-Hwan Ku, Do Young Kim, Ho-Yong Park

**Affiliations:** 1 Department of Biotechnology, KRIBB School of Bioscience, Korea University of Science and Technology (UST), Daejeon 34113, Korea; bl127@kribb.re.kr; 2 Industrial Bio-Materials Research Center, KRIBB, Daejeon 34141, Korea; kjh1018@kribb.re.kr (J.K.); sonkh@kribb.re.kr (K.-H.S.); 3 Insect Biotech Co. Ltd., Daejeon 34054, Korea; dhshin@insectbiotech.co.kr (D.-H.S.); balmania@insectbiotech.co.kr (B.-H.K.)

**Keywords:** *Cellulosimicrobium funkei*, GH64, endo-β-1,3-glucanase, RICIN, anti-fungal activity

## Abstract

Endo-β-1,3-glucanase plays an essential role in the deconstruction of β-1,3-d-glucan polysaccharides through hydrolysis. The gene (1650-bp) encoding a novel, bi-modular glycoside hydrolase family 64 (GH64) endo-β-1,3-glucanase (GluY) with a ricin-type β-trefoil lectin domain (RICIN)-like domain from *Cellulosimicrobium funkei* HY-13 was identified and biocatalytically characterized. The recombinant enzyme (rGluY: 57.5 kDa) displayed the highest degradation activity for laminarin at pH 4.5 and 40 °C, while the polysaccharide was maximally decomposed by its C-terminal truncated mutant enzyme (rGluYΔRICIN: 42.0 kDa) at pH 5.5 and 45 °C. The specific activity (26.0 U/mg) of rGluY for laminarin was 2.6-fold higher than that (9.8 U/mg) of rGluYΔRICIN for the same polysaccharide. Moreover, deleting the C-terminal RICIN domain in the intact enzyme caused a significant decrease (>60%) of its ability to degrade β-1,3-d-glucans such as pachyman and curdlan. Biocatalytic degradation of β-1,3-d-glucans by inverting rGluY yielded predominantly d-laminaripentaose. rGluY exhibited stronger growth inhibition against *Candida albicans* in a dose-dependent manner than rGluYΔRICIN. The degree of growth inhibition of *C. albicans* by rGluY (approximately 1.8 μM) was approximately 80% of the fungal growth. The superior anti-fungal activity of rGluY suggests that it can potentially be exploited as a supplementary agent in the food and pharmaceutical industries.

## 1. Introduction

β-1,3-d-Glucans are widely distributed in nature as a group of structural polysaccharides consisting of β-1,3-linked d-glucose residues in the cell walls of yeasts, filamentous fungi, algae, and plants [1]. Some bacteria have also been known to extracellularly produce curdlan, a linear β-1,3-d-glucan with different physicochemical properties [2,3]. These polysaccharides are generally decomposed by the concerted action of endo- and exo-type β-1,3-glucanases [4]. Of these enzymes, endo-β-1,3-glucanases (EC 3.2.1.39) are primary hemicellulolytic enzymes responsible for the decomposition of β-1,3-d-glucan polysaccharides and currently categorized into seven glycoside hydrolase (GH) families (16, 17, 50, 55, 64, 81, and 128) based on their primary structural similarities (http://www.cazy.org/Glycoside-Hydrolases.html) [5]. However, the majority of endo-β-1,3-glucanases are distributed between four GH families (16, 55, 64, and 81). The GH16 endo-β-1,3-glucanases are retaining enzymes with a β-jelly roll structure, while GH55, GH64, and GH81 endo-β-1,3-glucanases are inverting enzymes [6]. Due to their anti-fungal activity against pathogenic fungi, endo-β-1,3-glucanases have attracted a great deal of attention in the food and pharmaceutical industries as potential biocontrol agents [7,8]. In addition, these biocatalysts could be useful for the preparation of yeast extract [9], β-1,3-d-oligosaccharides as a potential immunoactivator [10], fungal cell protoplasts [11,12], etc.

So far, many herbivorous invertebrates and their symbiotic bacteria have been reported to produce diverse cellulases and hemicellulases with different substrate specificities [4,13]. Moreover, metagenomic analyses have shown that the intestinal microbiomes associated with herbivorous invertebrates include a specific carbohydrate-decomposing system [14,15]. Therefore, in this study, we identified the GluY gene encoding a bi-modular GH64 endo-β-1,3-glucanase from the whole genome sequence of *Cellulosimicrobium funkei* HY-13 KCTC 11302BP that was isolated from the intestine of the earthworm *Eisenia fetida*. *C. funkei* HY-13 is one of the representative fibrolytic gut bacteria and possesses at least six endo-type glycoside hydrolases showing peculiar biocatalytic activities toward cellulosic and hemicellulosic polysaccharides [16,17,18].

Here, we report the genetic and biochemical characteristics of a novel anti-fungal GH64 endo-β-1,3-glucanase (GluY) with a ricin-type β-trefoil lectin domain (RICIN)-like domain from *C. funkei* HY-13. The functional role of the RICIN domain in the decomposition of β-1,3-d-glucans is also described based on the substrate-binding capacities and degree of fungal inhibition of the two recombinant enzymes, rGluY and rGluYΔRICIN.

## 2. Materials and Methods

### 2.1. Chemicals

Curdlan from *Alcaligenes faecalis*, pachyman, ivory nut mannan, and a series of d-laminarioligosaccharides (L_2_–L_6_) were purchased from Megazyme International Ireland Ltd. (Wicklow, Ireland). All other chemical compounds, including *para*-nitrophenyl (*p*NP)-sugar derivatives, d-glucose, laminarin, lignin, Avicel PH-101, oat spelts xylan, beechwood xylan, barley β-1,3-1,4-d-glucan, and carboxymethylcellulose, were obtained from Sigma-Aldrich (St. Louis, MO, USA).

### 2.2. Cloning of the Endo-β-1,3-glucanase (GluY) Gene

For the overproduction of mature recombinant GluY proteins, PCR amplification of its encoding gene with an NdeI restriction site in the N-terminus and a HindIII restriction site in the C-terminus was conducted using the two oligonucleotide primers GluY-F (5′-CATATGGTCCCGGCGACCATCCCG-3′) and GluY-R (5′-AAGCTTTCAGAACGAC- CAGCGCTGGG-3′). The genomic DNA of *C. funkei* HY-13 was employed as a template and the reaction was performed using a PCR thermal cycler (TaKaRa), as described elsewhere [19]. The initial template denaturation was carried out for 4 min at 95 °C, followed by 35 cycles of 30 s at 95 °C, 30 s at 59.5 °C, and 1 min 40 s at 72 °C. The amplified PCR products were separated by electrophoresis on a 1.2% agarose gel, followed by purification of the desired gene products using a NucleoSpin Gel and PCR Clean-up (Macherey-Nagel, Düren, Germany). The purified gene products (1650-bp) were then cloned into a pGEM-T easy vector (Promega, Madison, WI, USA). The pGEM-T easy/*gluY* vectors were subsequently digested with NdeI and HindIII to generate *gluY* fragments with corresponding sticky ends. After purification of the resulting *gluY* fragments, they were cloned into a pET-28a(+) vector (Novagen, Darmstadt, Germany) with the same sticky ends and then the constructed pET-28a(+)/*gluY* vectors were transformed into *Escherichia coli* BL21. Similarly, the gene coding for GluYΔRICIN was amplified by PCR employing the following oligonucleotide primers: GluYΔRICIN-F (5′-CATATGGTCCCGGCGACCATCCCG-3′) and GluYΔRICIN-R (5′-AAGCTTTCAGCCACCGTCGTCGCCGAT-3′). In this case, the initial denaturation of template DNA was performed for 4 min at 95 °C, followed by 35 cycles of 30 s at 95 °C, 30 s at 59.5 °C, and 1 min 10 s at 72 °C. The amplified gene products (1119-bp) were purified and cloned into a pGEM-T easy vector (Promega, Madison, WI, USA), as described above. The pGEM-T easy/*gluYΔRICIN* vectors, which were transformed into *E. coli* DH5α, were isolated from the recombinant cells and digested with the aforementioned restriction enzymes to generate *gluYΔRICIN* fragments with the corresponding cohesive ends. After cloning of the purified *gluYΔRICIN* fragments into a pET-28a(+) vector (Novagen, Darmstadt, Germany), the resulting pET-28a(+)/*gluYΔRICIN* vectors were introduced into *E. coli* BL21.

### 2.3. Production and Purification of Recombinant Proteins

Overproduction of rGluY and rGluYΔRICIN was accomplished by cultivating the recombinant *E. coli* BL21 cells harboring pET-28a(+)/*gluY* or pET-28a(+)/*gluYΔRICIN* using a 5-L baffled flask, which included 1 L of Luria-Bertani broth (Duchefa Biochemie) and 25 mg/L of kanamycin, in a rotary shaker (150 rpm) for 12 h at 30 °C. The expression of the GluY and GluYΔRICIN genes was induced by the addition of 1 mM IPTG after the absorbance (*A*_600_) of the cultures at 600 nm reached 0.4–0.5. The rGluY- or rGluYΔRICIN-expressing cells were isolated from the culture broth by centrifugation (5000× *g*) for 20 min at 4 °C, after which they were resuspended in the binding buffer [18]. After disruption of the recombinant cells by sonication, the rGluY proteins produced as inactive inclusion bodies were collected by centrifugation (12,000× *g*) for 20 min at 4 °C. On the other hand, rGluYΔRICIN proteins were recovered in an active form in the soluble fraction. Thus, after solubilization of the collected inclusion bodies in binding buffer (20 mM Tris-HCl (pH 8.0), 0.5 M NaCl, 5 mM imidazole, 1 mM 2-mercaptoethanol, and 6 M guanidine hydrochloride), the inactive rGluY proteins were purified in an active form by on-column refolding using a HisTrap HP (GE Healthcare, Uppsala, Sweden) (5 mL) column attached to a fast-protein liquid chromatography system (Amersham Pharmacia Biotech, Uppsala, Sweden), according to the protocols provided by the manufacturer. The active rGluYΔRICIN proteins were simply isolated by affinity column chromatography using the same HisTrap HP (GE Healthcare, Uppsala, Sweden) (5 mL) column, according to the manufacturer’s instructions. In both cases, the recombinant enzymes were eluted from the column using a linear gradient of 20–500 mM imidazole at a flow rate of 2 mL/min. The fractions with high endo-β-1,3-glucanase activity were selectively collected and combined, followed by desalting with a HiPrep 26/10 desalting column (GE Healthcare, Uppsala, Sweden) using 50 mM sodium phosphate buffer (pH 6.0) as the mobile phase. The active fractions were recovered and subjected to further analysis.

### 2.4. Analysis of Proteins

Analysis of the relative molecular masses of purified rGluY and rGluYΔRICIN was performed by sodium dodecyl sulfate-polyacrylamide gel electrophoresis (SDS-PAGE) of the denatured proteins in a 12.0% gel. The protein bands separated in the gels were visualized by staining with Coomassie Brilliant Blue R-250. The protein concentrations were determined by Bradford assay employing bovine serum albumin as a standard.

### 2.5. Enzyme Assays

Endo-β-1,3-glucanase activity was assayed by quantitatively determining the amount of reducing sugars released after enzymatic hydrolysis of laminarin using 3,5-dinitrosalicylic acid (DNS) reagent and glucose as a standard [20]. The standard reaction mixture (0.5 mL) was composed of 1.0% laminarin and an appropriately diluted enzyme solution (0.05 mL) in 50 mM sodium citrate buffer (pH 5.5). The enzyme reaction was conducted at 45 °C for 20 min. After finishing the biocatalytic reaction, the DNS reagent (0.75 mL) was inserted into the reaction mixture, followed by heating at 100 °C for 5 min to develop the red-brown color. One unit (U) of endo-β-1,3-glucanase activity toward β-1,3-d-glucan polysaccharides was defined as the amount of protein required to produce 1 μmol of reducing sugar per min under standard assay conditions.

### 2.6. Effects of pH, Temperature, and Chemicals on the Endo-β-1,3-glucanase Activity

The effect of pH on the endo-β-1,3-glucanase activities of rGluY and rGluYΔRICIN was assessed by exposing samples to pH values ranging from 3.5 to 11.0 at 45 °C for 20 min using the following buffer systems (50 mM): sodium citrate (pH 3.5–5.5), sodium phosphate (pH 5.5–7.5), Tris-HCl (pH 7.5–9.0), and glycine-NaOH (pH 9.0–11.0). In this case, the laminarin-degrading activity of respective enzyme was approximately 5 U/mL at an optimum pH. To examine the pH stabilities of the two recombinant enzymes, they were first pre-incubated in various pH buffers for 1 h at 4 °C, followed by measurements of their residual endo-β-1,3-glucanase activities. In this experiment, the biocatalytic reaction was started by adding 1.0% laminarin to the reaction mixture. The temperature optima of rGluY and rGluYΔRICIN were evaluated by reacting each recombinant enzyme with laminarin at 25, 30, 35, 40, 45, 50, 55, and 60 °C in 50 mM sodium citrate buffer (pH 5.5). In this case, the laminarin-hydrolyzing activity of respective enzyme was approximately 5 U/mL at an optimum temperature. Meanwhile, the thermostabilities of the two recombinant enzymes were estimated by assaying their residual endo-β-1,3-glucanase activities after pre-incubation of the biocatalysts at the corresponding temperature for 1 h in 50 mM sodium citrate buffer (pH 5.5). The effects of divalent cations (each 1 mM) and chemical compounds (each 5 mM) on the endo-β-1,3-glucanase activity of rGluY was evaluated after pre-incubation of the biocatalyst (5 U/mL) at 4 °C for 10 min in 50 mM sodium citrate buffer (pH 5.5) including the compound of interest.

### 2.7. Analysis of the Degradation Products

Biocatalytic degradation of β-1,3-d-glucans (each 1 mg) including laminarin, pachyman, and curdlan together with d-laminarioligosaccharides (L_2_–L_6_, each 1 mg) was carried out by incubating the substrates with rGluY (1 μg) in 0.5 mL of 50 mM sodium citrate buffer (pH 5.5) at 45 °C for 12 h, during which time the biocatalysts remained fairly stable. After stopping the enzyme reaction by boiling for 5 min, the degradation products were analyzed by thin-layer chromatography (TLC) and high performance liquid chromatography (HPLC) employing d-glucose (G_1_) and a series of d-laminarioligosaccharides (L_2_–L_6_) as standards. TLC was carried out using a silica gel 60 F254 plate (20 × 20 cm, Merck) with the developing solvent system consisting of 1-butanol, acetic acid, and water at a ratio of 2:1:1. The products were visualized by spraying the dried TLC plate with ethanol/sulfuric acid (95:5, *v*/*v*) and heating at 100 °C for 10 min. HPLC analysis was performed using a Finnigan Surveyor Modular HPLC systems (Thermo Electron Co., Waltham, MA, USA) equipped with an Asahipak NH2P-50 4E column (5 μm, 4.6 × 250 mm, Shodex). A mixture of acetonitrile and water at a ratio of 6:4 was used as a mobile phase and the flow rate was 1 mL/min.

### 2.8. Binding Assay

Substrate-binding capacities of rGluY and rGluYΔRICIN were examined by employing insoluble polymeric materials with different microstructures, such as curdlan, avicel PH-101, oat spelts xylan, ivory nut mannan, and lignin. To remove any water-soluble substances before the binding assay, the candidate polymers were carefully washed four times with sterile distilled water, followed by rewashing with 50 mM sodium citrate buffer (pH 5.5). The binding abilities of the recombinant enzymes to the insoluble materials were then investigated as follows. The appropriately diluted enzyme preparation (5.0 U/mL) plus an equal volume of hydrophobic polymer was firstly incubated in a 1.5 mL Eppendorf tube on ice for 2 h being vigorously stirred every 5 min. Subsequently, the supernatants containing rGluY or rGluYΔRICIN unbound to the substrate polymers were carefully recovered by centrifugation (12,000× *g*) and applied directly to the determination of protein concentration and remaining endo-β-1,3-glucanase activity.

### 2.9. Anti-Fungal Assay

*Candida albicans* KCTC 7965 was cultivated in 30 mL of YPD medium (1.0% yeast extract, 2.0% peptone, and 2.0% dextrose) at 30 °C for 24 h. The yeast cells were then harvested by centrifugation (12,000× *g*) at 4 °C for 20 min, after which the cell pellet was resuspended in 1 mL of sterile distilled water to an *A*_600_ of 0.5. Prior to the anti-fungal assay, the cell suspension was further diluted 10^4^ times with sterile distilled water. An aliquot of the diluted cell suspension (80 μL) was then mixed with various concentrations (0.05, 0.1, 0.2, and 0.5 mg/mL) of purified rGluY (20 μL) or rGluYΔRICIN (20 μL) in 50 mM sodium citrate buffer (pH 5.5). To induce degradation of the *C. albicans* cell wall by the recombinant endo-β-1,3-glucanases, the mixtures were preincubated at 37 °C for 30 min and thereafter they were spread out on YPD agar plates, followed by incubating at 30 °C for 20 h. The degree of fungal growth inhibition by rGluY and rGluYΔRICIN was assessed by counting the number of colonies formed on YPD agar plates.

## 3. Results and Discussion

### 3.1. Molecular Characterization of the GH64 Endo-β-1,3-glucanase Gene

**Figure 1:**
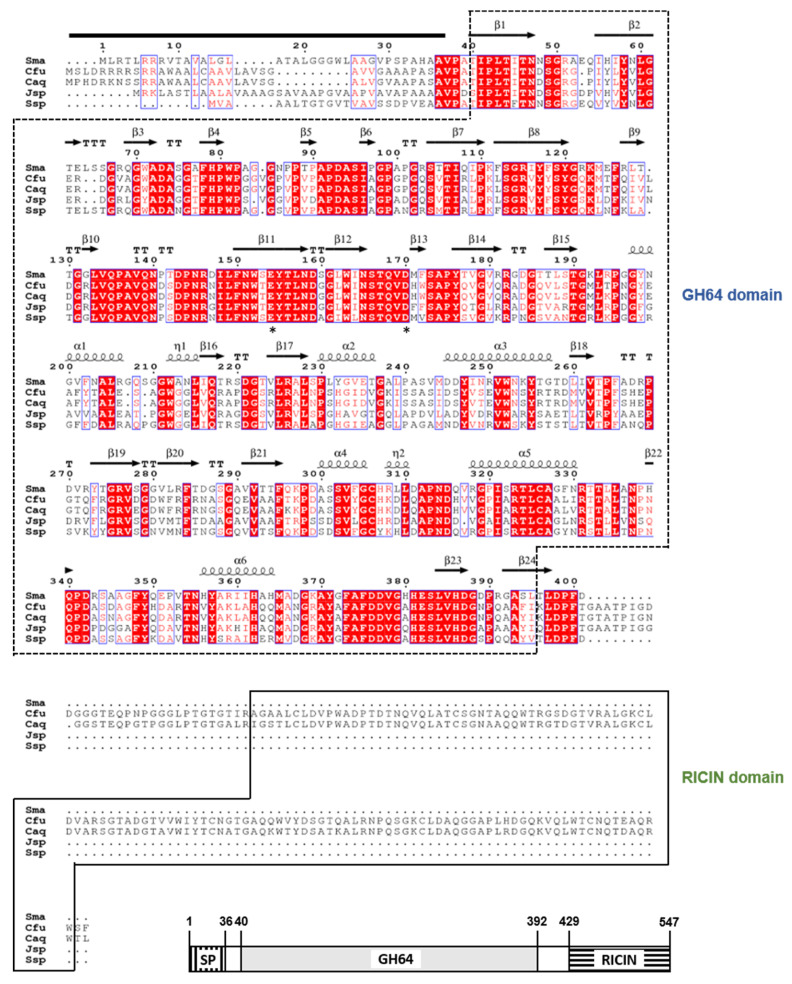
Domain architecture of *Cellulosimicrobium funkei* HY-13 GH64 endo-β-1,3-glucanase and structure-based sequence alignment of the enzyme and its structural homologs. The first line displays the secondary structure elements (α-helix, squiggle; β-strand, arrow; 3_10_-helix, η; β-turn, TT) of *Streptomyces matensis* DIC-108 endo-β-1,3-glucanase (PDB code: 3GD0) employed as a template. Sequences (GenBank accession numbers): Sma, *Streptomyces matensis* DIC-108 endo-β-1,3-glucanase (BAA34349); Cfu, *C. funkei* HY-13 endo-β-1,3-glucanase (MT332201); Caq, *Cellulosimicrobium aquatile* endo-β-1,3-glucanase (WP_083711238); Jsp, *Jiangella* sp. sugar hydrolase (WP_092617602); and Ssp, *Streptomyces* sp. sugar hydrolase (KOX25504). The predicted signal peptide is indicated by a black bar. GH64 and RICIN domains are outlined by dotted and solid lines, respectively. Strictly conserved amino acid residues that play an essential role in biocatalysis are shown by asterisks.

The 1650-bp GluY gene (GenBank accession number: MT332201) identified from the complete genome sequence of *C. funkei* HY-13 was predicted to encode a protein of 549 amino acids, which has a calculated pI of 6.00 and a deduced molecular mass of 58,232 Da. Also, the premature GluY was evaluated to be an extracellular protein with a signal peptide that might be cleaved between Ala36 and Val37 in the N-terminus region, as predicted by the SignalP 5.0 server (http://www.cbs.dtu.dk/services/SignalP/) (Figure 1). Compared to the premature GluY, the mature GluY without an N-terminal signal peptide was identified to be a polypeptide with a calculated pI of 5.43 and a deduced molecular mass of 54,611 Da. Pfam and protein BLAST analyses of the primary sequence of GluY indicated that it might be a bi-modular GH enzyme composed of an N-terminal catalytic GH64 domain (from Thr40 to Ile392) and a C-terminal RICIN domain (from Ala429 to Trp547). Recently, RICIN domain has been demonstrated to participate in the positive regulation of enzyme-substrate binding [21]. A protein BLAST survey showed that the domain architecture of GluY was most similar to that of an uncharacterized GH64 endo-β-1,3-glucanase (WP_154799449) identified through a whole genome survey of *Cellulosimicrobium* sp. BI34T. It was predicted that the GluYΔRICIN gene (1119-bp) encoded a polypeptide (from Val37 to Gly409) of 373 amino acids with a calculated pI of 5.52 and a deduced molecular mass of 39,962 Da.

**Figure 2:**
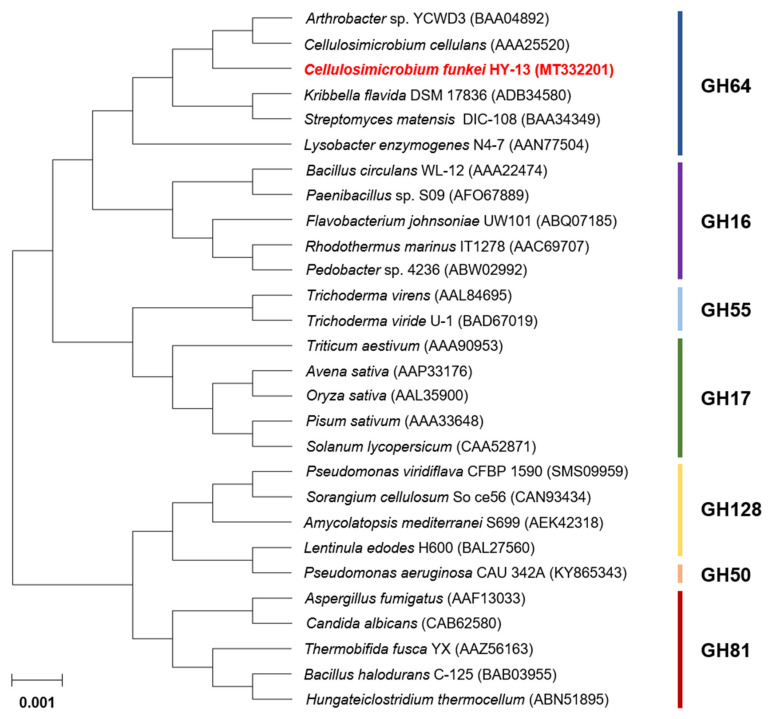
Phylogenetic tree of *C. funkei* HY-13 GH64 endo-β-1,3-glucanase (GluY) and its closely related functional homologs. Alignment of the amino acid sequences was performed using ClustalW in the MegAlign program (DNASTAR Inc. (Madison, WI, USA)). The protein sequences employed for phylogenetic analysis were retrieved from the GenBank database.

Multiple sequence alignment exhibited that the catalytic GH64 domain (from Thr40 to Ile392) of premature GluY was 97% and 93% identical to that of *Cellulosimicrobium aquatile* glucan 1,3-β-glucosidase (GenBank accession number: WP_083711238) and *Cellulosimicrobium cellulans* glucan 1,3-β-glucosidase (WP_115941918), which had not been functionally characterized but were only identified through a genome survey. In addition, the catalytic GH64 domain of premature GluY shared 64% and 63% sequence identities with that of *Promicromonosporaceae bacterium* CFH30434 glucan 1,3-β-glucosidase (WP_125776063) and *Streptomyces* sp. NRRL F-6491 sugar hydrolase (KOX25504), respectively. The phylogenetic analysis also revealed that the primary structure of premature GluY resembled that of members within GH family 64, which is made up of inverting endo-β-1,3-glucanases (Figure 2). Meanwhile, sequence identities between the RICIN domain (from Ala429 to Trp547) of premature GluY and that of the uncharacterized *Cellulosimicrobium* sp. BI34T glucan 1,3-β-glucosidase (WP_154799449) and *C. aquatile* glucan 1,3-β-glucosidase (WP_083711238) were evaluated to be 96% and 91%, respectively. However, the biocatalytic characteristics of GH64 endo-β-1,3-glucanases having a RICIN domain have not been well documented, although some of their encoding genes were identified and deposited in the NCBI database. Only two GH64 endo-β-1,3-glucanases with a RICIN domain from *Arthrobacter* sp. strain YCWD3 [22] and *Oerskovia xanthineolytica* (Recently *Cellulosimicrobium cellulans*) [23], which share 99% amino acid sequence identity [24], have been reported to be partially characterized to date. The two conserved residues, Asp169 acting as the catalytic nucleophile/base and Glu153 acting as the catalytic proton donor, were observed in the active site of premature GluY, as shown in other GH64 endo-β-1,3-glucanases [6].

The secondary structure elements of GluY from *C. funkei* HY-13, which were predicted employing a GH64 endo-β-1,3-glucanase from *Streptomyces matensis* DIC-108 (PDB code: 3GD0) as a template, are shown in Figure 1. The structure-based sequence alignment rendered using ESPript software 3.0 (https://espript.ibcp.fr/ESPript/ESPript/) revealed that the catalytic GH64 domain in GluY from *C. funkei* HY-13 consisted of 6 α-helices, 24 β-strands, 2 3_10_-helices, and 13 β-turns.

### 3.2. Purification and SDS-PAGE Analysis of Recombinant Enzymes

When overexpressed in *E. coli* BL21, the majority of rGluY proteins were produced as insoluble inclusion bodies, while rGluYΔRICIN was produced as a soluble protein with endo-β-1,3-glucanase activity similar to other non-modular GH64 enzymes [24,25]. It is believed that the formation of rGluY inclusion bodies might be due to the C-terminal RICIN domain exhibiting relatively high hydrophobicity. Based on the protein solubility, active rGluY proteins were purely isolated to electrophoretic homogeneity by an on-column protein refolding method employing a His-tag column. In a much simpler process, rGluYΔRICIN was purified by basic affinity chromatography using the same column.

**Figure 3:**
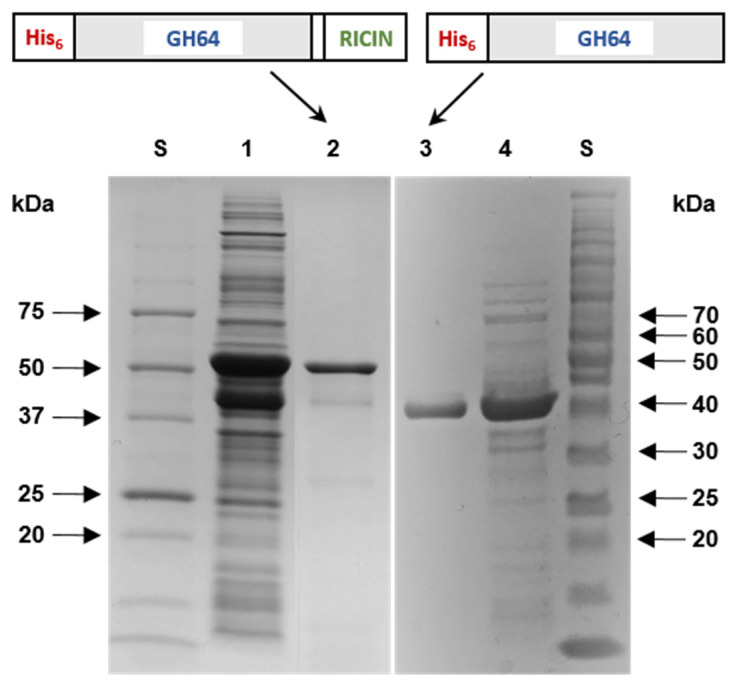
SDS-PAGE of the purified rGluY and rGluYΔRICIN after affinity chromatography on HisTrap HP. Lane S, standard marker proteins; lane 1, the whole cell lysate of rGluY-expressing *E. coli* BL21 after IPTG induction; lane 2, purified rGluY; lane 3, purified rGluYΔRICIN; lane 4, the soluble cell lysate of rGluYΔRICIN-expressing *E. coli* BL21 after IPTG induction.

The relative molecular masses of purified rGluY and rGluYΔRICIN were evaluated to be approximately 57.5 kDa and 42.0 kDa, respectively, as analyzed by SDS-PAGE (Figure 3). These values agreed well with the deduced molecular masses of rGluY (57,274 Da) and rGluYΔRICIN (42,783 Da), which were determined by the Compute pI/MW server (https://www.expasy.org/resources/compute-pi-mw). The molecular size (57.5 kDa) of rGluY with a RICIN domain was larger than that (39.4 kDa) of a non-modular GH64 endo-β-1,3-glucanase from *Streptomyces matensis* DIC-108 [6], that (41.0 kDa) of a non-modular GH64 endo-β-1,3-glucanase from *Lysobacter enzymogenes* N4-7 [25], and that (43.0 kDa) of a non-modular GH64 endo-β-1,3-glucanase from *Kribbella flavida* NBRC 14,399 [26] (Table 1). However, the molecular mass (57.5 kDa) of the enzyme was similar to that (55.0 kDa) of a modular GH64 endo-β-1,3-glucanase from *Arthrobacter* sp. strain YCWD3 [22].

### 3.3. Biocatalytic Characterization of Recombinant Enzymes

Similar to the non-modular GH64 endo-β-1,3-glucanase (GluB) from *L. enzymogenes* [25], the highest endo-β-1,3-glucanase activity of rGluY for laminarin was observed when the biocatalytic reaction was performed at 40 °C in 50 mM sodium citrate buffer (pH 4.5) (Table 1). However, the biocatalytic capacity of rGluY was remarkably reduced at pH values below 4.0 (<30% of its maximum activity) or above 8.5 (<65% of its maximum activity), and at temperatures over 50 °C (<65% of its maximum activity). The optimum pH and temperature of rGluY was comparable to a GH64 endo-β-1,3-glucanase from *S. matensis* DIC-108, which was most active toward curdlan at 55 °C in pH range 7.5–8.5 [6]. Compared to rGluY, rGluYΔRICIN displayed a maximum degradation activity for laminarin at 45 °C and pH 5.5, similar to the GH64 endo-β-1,3-glucanase (KfGH64) from *Kribbella flavida* NBRC 14,399 [26]. Additionally, the endo-β-1,3-glucanase activity of the C-terminal truncated mutant enzyme was sharply decreased at pH values less than 4.0 (<30% of its maximum activity) or more than 5.5 (<60% of its maximum activity), and at temperatures exceeding 50 °C (<65% of its maximum activity). It is believed that the significant decrease in rGluYΔRICIN activity at pH values above pH 5.5 might be attributed to the deletion of a RICIN domain in GluY. In this study, rGluY and rGluYΔRICIN were relatively stable in a broad pH range (3.5–10.5) because these enzymes retained more than 85% of their original endo-β-1,3-glucanase activity at those pH values even when exposed at 4 °C for 1 h in the absence of laminarin. Moreover, the two recombinant enzymes were fairly stable at temperatures below 45 °C for 1 h, but their thermostabilities were drastically downregulated in a temperature-dependent manner when exposed to temperatures exceeding 45 °C for the same preincubation period. The present results implied that the deletion of the RICIN domain in GluY did not induce any notable alteration in the pH stability and thermostability of rGluY.

**Figure 4:**
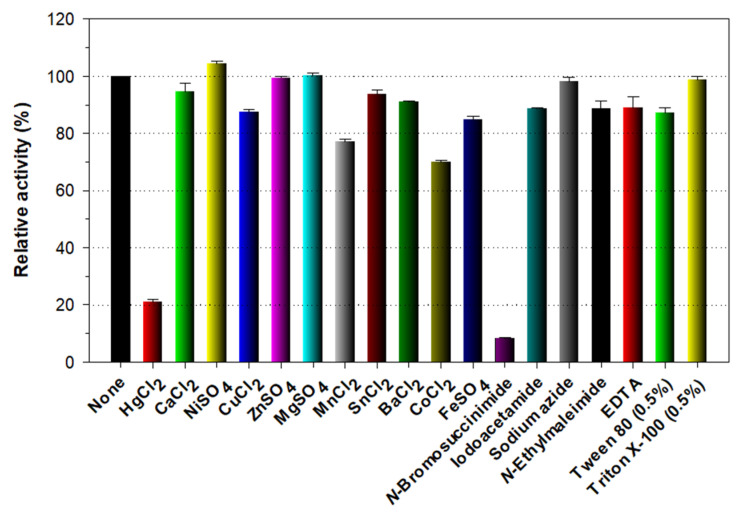
Effects of metal ions (1 mM) and chemical reagents (5 mM) on the endo-β-1,3-glucanase activity of rGluY. The values are mean ± SD of triplicate tests.

When the enzyme reactions were conducted in the presence of tryptophan (Trp) residue-specific modifiers including Hg^2+^ (1 mM) and *N*-bromosuccinimide (5 mM), rGluY was considerably inactivated by the compounds (Figure 4). Previously, a similar observation was also made with a GH64 endo-β-1,3-glucanase from *S. matensis* DIC-108 [6] reacted with curdlan in the presence of Hg^2+^ (>20 μM), although the biocatalytic activity of *Laceyella putida* GH16 endo-β-1,3-glucanase [27] was only moderately suppressed by the metal ion. Taken together, the findings were consistent with the fact that *N*-bromosuccinimide and Hg^2+^ ions oxidize the indole ring of strictly conserved Trp residues in the active site of endo-type GH enzymes, which essentially participate in enzyme-substrate interaction [28,29]. The endo-β-1,3-glucanase activity of rGluY was not substantially affected by EDTA (5 mM), Tween 80 (0.5%), or divalent cations (each 1 mM) such as Ca^2+^, Ni^2+^, Cu^2+^, Zn^2+^, Mg^2+^, Sn^2+^, Ba^2+^, and Fe^2+^. The exceptions were Mn^2+^ and Co^2+^, which resulted in 23% and 30% reductions in its original biocatalytic activity, respectively (Figure 4). Conversely, it was recently found that the biocatalytic activity a GH64 endo-β-1,3-glucanase with a βγ-crystalline domain from *Clostridium beijerinckii* was greatly increased by >2-fold in the presence of 1 mM Ca^2+^ [30]. Moreover, the biocatalytic activity of *Paenibacillus* sp. endo-β-1,3-glucanase has been shown to be down-regulated by 37% in the presence of 2 mM Zn^2+^ [31].

### 3.4. Substrate Specificity

Using various cellulosic and hemicellulosic polysaccharides with a specific microstructure and *p*NP-sugar derivatives, the substrate specificities of rGluY and rGluYΔRICIN were investigated (Table 2). Of the evaluated polymeric materials, the two enzymes were capable of preferentially degrading β-1,3-d-glucans with the following order: laminarin > pachyman > curdlan. On the other hand, they did not exhibit any detectable biocatalytic activities toward *p*NP-sugar derivatives or other polysaccharides made up of d-glucose, d-mannose, or d-xylose molecules linked by β-1,4-glycosidic bonds in the backbone. Taken together, these results clearly indicated that rGluY and its C-terminal truncated mutant enzyme were true endo-β-1,3-glucanases without other carbohydrolase activities. The specific activity of rGluY toward laminarin, pachyman, and curdlan was measured to be 26.0 U/mg, 18.8 U/mg, and 2.3 U/mg, respectively. However, deleting the C-terminal RICIN domain in the enzyme gave rise to a significant decrease (>60%) of its ability to decompose the aforementioned β-1,3-d-glucan polysaccharides. In this case, the specific activity of rGluYΔRICIN for laminarin, pachyman, and curdlan was assessed to be 9.8 U/mg, 5.4 U/mg, and 0.4 U/mg, respectively. As listed in Table 1, the biocatalytic activity (26.0 U/mg) of rGluY for laminarin was approximately 6.2- and 12.4-fold, respectively, higher than that (approx. 4.2 U/mg) of *S. matensis* DIC-108 GH64 endo-β-1,3-glucanase [6] and that (2.1 U/mg) of *L. enzymogenes* N4-7 GH64 endo-β-1,3-glucanase [25] for the same polysaccharide. Likewise, rGluY (18.8 U/mg) was evaluated to be the most active on pachyman compared to other characterized GH64 functional homologs including *Arthrobacter* sp. strain YCWD3 endo-β-1,3-glucanase (11~13 U/mg) [22] and *L. enzymogenes* N4-7 GH64 endo-β-1,3-glucanase (approx. 0.6 U/mg) [25]. It should also be noted that the degradation activity (2.3 U/mg) of rGluY for curdlan was approximately three-fold lower than that (7.1 U/mg) of *S. matensis* DIC-108 GH64 endo-β-1,3-glucanase [6] for the same substrate. However, the curdlan-degrading activity (2.3 U/mg) was relatively similar to that (1.2 U/mg) of *L. enzymogenes* N4-7 GH64 endo-β-1,3-glucanase [25]. Based on these findings, it is proposed that rGluY is a novel β-1,3-d-glucan-degrading biocatalyst exhibiting different substrate specificities, which is distinguished from other known GH64 enzymes (Table 1).

**Figure 5:**
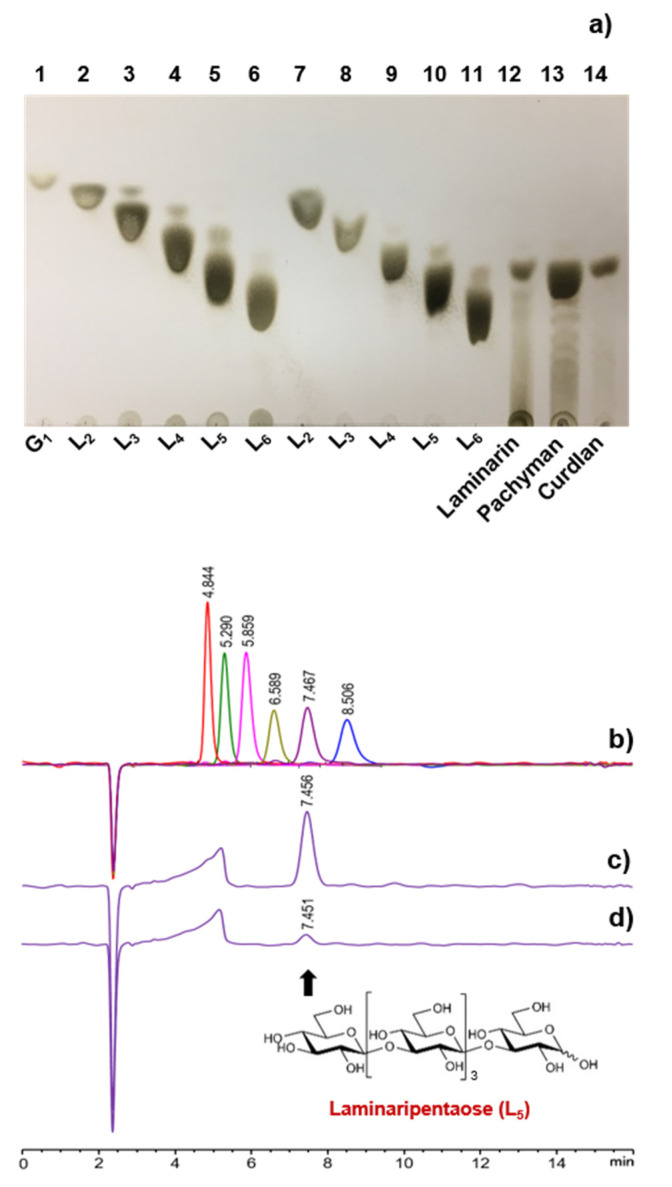
TLC and HPLC analyses of degradation products of d-laminarioligosaccharides (L_2_–L_6_) and β-1,3-d-glucans by rGluY: (**a**) Lanes 1 to 6, standard markers (d-glucose, G_1_; d-laminaribiose, L_2_; d-laminaritriose, L_3_; d-laminaritetraose, L_4_; d-laminaripentaose, L_5_; d-laminarihexaose, L_6_); lanes 7 to 11, reaction of rGluY with d-laminarioligosaccharides (L_2_–L_6_, each 1 mg); lane 12, reaction of rGluY with 1 mg laminarin; lane 13, reaction of rGluY with 1 mg pachyman; lane 14, reaction of rGluY with 1 mg curdlan; (**b**) total ion chromatogram of the standards (G_1_, a peak with a retention time (RT) of 4.844; L_2_, a peak with a RT of 5.290; L_3_, a peak with a RT of 5.859; L_4_, a peak with a RT of 6.589; L_5_, a peak with a RT of 7.467; L_6_, a peak with a RT of 8.506); (**c**) total ion chromatogram of the degradation products of pachyman; (**d**) total ion chromatogram of the degradation products of curdlan.

Unlike some endo-β-1,3-glucanases belonging to different GH families [5,32,33], it has been previously shown that *S. matensis* DIC-108 GH64 endo-β-1,3-glucanase (LPHase) exclusively degrades insoluble curdlan to d-laminaripentaose (L_5_) [24]. Similarly, *S. matensis* ATCC 23,935 GH64 endo-β-1,3-glucanase (SmβG) was also reported to preferentially hydrolyze curdlan to L_5_ (79%) as the major end product together with L_2_–L_4_ and other soluble d-laminarioligosaccharides with degree of polymerization > 5 [8]. Thus, we investigated the degradation patterns of *C. funkei* HY-13 GH64 endo-β-1,3-glucanase (rGluY) toward d-laminarioligosaccharides (L_2_–L_6_) and β-1,3-d-glucans such as laminarin, pachyman, and curdlan. The results of TLC analysis clearly showed that a series of d-laminarioligosaccharides of L_2_ to L_6_ was not susceptible to rGluY (Figure 5a). However, the enzyme could readily degrade the evaluated β-1,3-d-glucans to predominantly produce L_5_ as the final product. In this case, only small amounts of d-laminarioligosaccharides with degree of polymerization > 5 were also detected as end products, while the production of d-laminarioligosaccharides of L_2_ to L_4_ by rGluY from the substrates was negligible or not detectable. Similarly, the results of HPLC analysis revealed that rGluY was most likely to predominantly produce L_5_ as the final product when reacted with pachyman and curdlan (Figure 5c,d). It seemed that with the exception of L_5_, the production of other d-laminarioligosaccharides by rGluY from the β-1,3-d-glucans was insignificant. Based on these results, it is suggested that rGluY is a new L_5_-releasing GH64 endo-β-1,3-glucanase exhibiting slightly different substrate degradation patterns, which is distinct from *S. matensis* DIC-108 GH64 endo-β-1,3-glucanase [24] and *S. matensis* ATCC 23,935 GH64 endo-β-1,3-glucanase [8].

### 3.5. Binding Affinity of rGluY and rGluYΔRICIN to Insoluble Substrates

**Figure 6:**
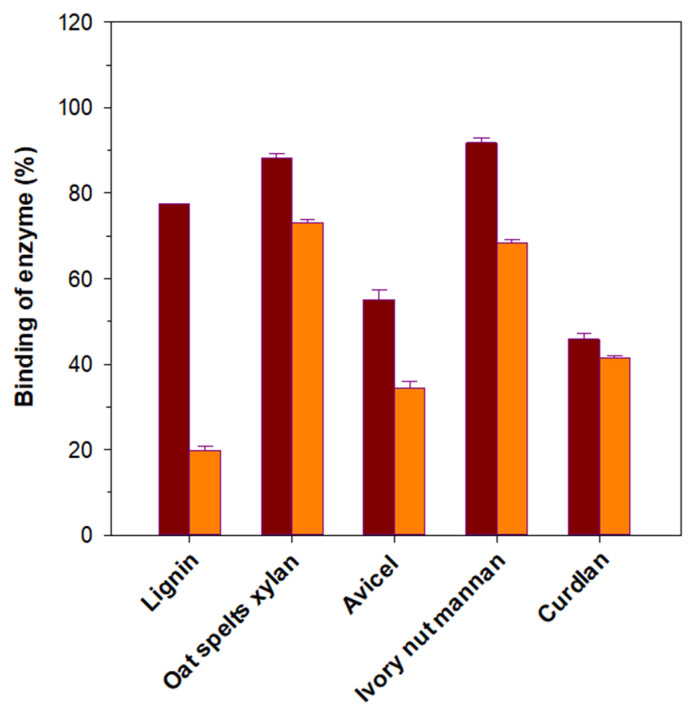
Binding of rGluY (■) and rGluYΔRICIN (■) to insoluble polymers. The values are mean ± SD of triplicate tests.

Though the functional role of a RICIN domain in *Luteimicrobium xylanilyticum* HY-24 GH10 endo-β-1,4-xylanase was recently demonstrated [21], the biological functions of a RICIN domain in microbial endo-β-1,3-glucanases in enzyme-substrate binding and biocatalysis have yet to be fully investigated. Thus, in this study, we examined the role of a C-terminal RICIN domain in GluY in enzyme-substrate interaction by comparing the substrate-binding capacities of rGluY and rGluYΔRICIN to insoluble polymers such as curdlan, Avicel PH-101, ivory nut mannan, oat spelts xylan, and lignin. It is of great interest to note that the binding ability of rGluY to lignin was significantly higher than that of rGluYΔRICIN to the same substrate polymer (Figure 6). Likewise, compared to the latter, the former displayed higher substrate-binding capacities to the same hydrophobic materials in the order of ivory nut mannan > oat spelts xylan > Avicel > curdlan. These substrate-binding patterns of rGluY and rGluYΔRICIN agreed well with the finding that a RICIN domain in *L. xylanilyticum* HY-24 GH10 endo-β-1,4-xylanase plays an important role in the enzyme-substrate interaction as a substrate-binding motif [21]. Moreover, similar to rGluYΔRICIN, *Paenibacillus* sp. GH16 endo-β-1,3-glucanase has been reported to exhibit reduced substrate-binding affinities to Avicel and curdlan when its four carbohydrate binding modules (CBMs) were removed from the intact enzyme [7].

### 3.6. Anti-Fungal Activities of rGluY and rGluYΔRICIN

**Figure 7:**
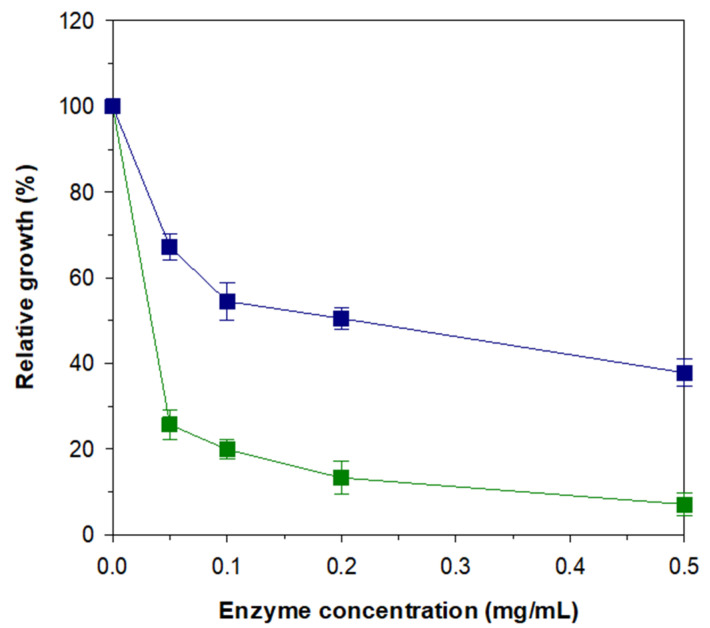
Inhibitory effects of rGluY (■) and rGluYΔRICIN (■) on the growth of *C. albicans*. The values are mean ± SD of triplicate tests.

Recently, endo-β-1,3-glucanases have drawn growing attention as a potential biocontrol agent, which can efficiently inhibit the growth of diverse fungal pathogens via cell wall degradation, in the fields of pharmaceuticals and food applications [7,8]. In this study, to elucidate the functional role of a C-terminal RICIN domain in GluY on fungal growth inhibition, we evaluated the anti-fungal activities of rGluY and its C-terminal truncated mutant enzyme against *C. albicans*. Figure 7 clearly shows that both rGluY and rGluYΔRICIN could substantially inhibit the growth of *C. albicans* in a dose-dependent manner. The degree of growth inhibition of *C. albicans* by rGluY at a concentration of 0.1 mg/mL (approximately 1.8 μM) was assessed to be approximately 80% of the fungal growth. Moreover, the enzyme was able to efficiently inhibit more than 90% of the fungal growth when used at a concentration of 0.5 mg/mL (approx. 9.0 μM). These results indicated that the anti-fungal activity of rGluY against *C. albicans* was relatively stronger than those of other known functional homologs against the same fungal pathogen. Previously, *S. matensis* ATCC 23,935 GH64 endo-β-1,3-glucanase (SmβG) [8] and *Thermotoga marimata* endo-β-1,3-glucanase (TmβG) [34] were also reported to inhibit over 60% of the growth of *C. albicans* at concentrations exceeding 0.1 mg/mL (approximately 2 μM) and 0.2 mg/mL (approximately 2.7 μM), respectively. Compared to rGluY, it was found that rGluYΔRICIN even at a concentration of 0.5 mg/mL (approximately 11.6 μM) could only inhibit 60% of the growth of *C. albicans*. Therefore, the stronger growth inhibition of *C. albicans* by rGluY can most likely be attributed to its C-terminal RICIN domain, which further supports preferential binding between the enzyme and the cell wall of *C. albicans*.

## 4. Conclusions

The bi-modular GH64 endo-β-1,3-glucanase (GluY) with a C-terminal RICIN domain from *C. funkei* HY-13 is a novel d-laminaripentaose-releasing enzyme with molecular and biochemical characteristics that is distinct from other previously characterized GH64 functional homologs. Compared to rGluYΔRICIN, rGluY showed higher degradation activity toward laminarin, pachyman, and curdlan, as well as higher substrate-binding affinity to diverse insoluble polysaccharides, indicative of the functional roles of the C-terminal RICIN domain in the enzyme. Considering its ability to strongly inhibit the growth of *C. albicans* in a dose-dependent manner, rGluY can be exploited as an effective anti-fungal agent for food and pharmaceutical applications.

## Figures and Tables

**Table 1:** Enzymatic features of bacterial GH64 endo-β-1,3-glucanases active for β-1,3-d-glucans.

Strain	Enzyme	M*_r_* (kDa)	Optimum pH	Optimum Temp. (°C)	Specific Activity (U/mg)	Reference
*Cellulosimicrobium funkei* HY-13	rGluY	57.5	4.5	40	26.0 ^a^, 18.8 ^b^, 2.3 ^c^	This study
*Cellulosimicrobium funkei* HY-13	rGluYΔRICIN	42.0	5.5	45	9.8 ^a^, 5.4 ^b^, 0.4 ^c^	This study
*Streptomyces matensis* DIC-108	LPHase	39.4	7.5–8.5	55	4.2 ^a^, 7.1 ^c^	[6]
*Lysobacter enzymogenes* N4-7	GluB	41.0	4.5–5.0	41	2.1 ^a^, 0.6 ^b^, 1.2 ^c^	[25]
*Streptomyces matensis* ATCC 23935	SmβG	43.0	6.0	55	NI ^d^	[8]
*Arthrobacter* sp. strain YCWD3	endo-β-1,3-glucanase	55.0	5.8	NI	11–13 ^b^	[22]
*Kribbella flavida* NBRC 14399	KfGH64	43.0	5.5	45	NI	[26]

^a^ Specific enzyme activity toward laminarin; ^b^ Specific enzyme activity toward pachyman; ^c^ Specific enzyme activity toward curdlan; ^d^ Not indicated.

**Table 2:** Biocatalytic activity of rGluY and rGluYΔRICIN toward different polysaccharides.

Substrate	Specific Activity (U/mg) ^a^ of rGluY	Specific Activity (U/mg) of rGluYΔRICIN
Laminarin	26.0 ± 1.2	9.8 ± 0.1
Pachyman	18.8 ± 1.4	5.4 ± 0.1
Curdlan	2.3 ± 0.2	0.4 ± 0.1
Carboxymethylcellulose	ND ^b^	ND
Barley β-1,3-1,4-d-glucan	ND	ND
Beechwood xylan	ND	ND
Locust bean gum	ND	ND

The values are mean ± SD of triplicate tests; ^a^ Specific activity was obtained from the three repeated experiments; ^b^ Not detected.

## Data Availability

Not applicable.
